# Electro‐Microfluidic Assembly Platform for Manipulating Colloidal Structures inside Water‐in‐Oil Emulsion Droplets

**DOI:** 10.1002/advs.202203341

**Published:** 2022-09-28

**Authors:** Shitao Shen, Xiaofeng Qin, Haoqiang Feng, Shuting Xie, Zichuan Yi, Mingliang Jin, Guofu Zhou, Eser Metin Akinoglu, Paul Mulvaney, Lingling Shui

**Affiliations:** ^1^ International Joint Laboratory of Optofluidic Technology and System National Centre for International Research on Green Optoelectronics South China Academy of Advanced Optoelectronics & School of Information and Optoelectronic Science and Engineering South China Normal University Guangzhou 510006 P. R. China; ^2^ International Academy of Optoelectronics at Zhaoqing South China Normal University Zhaoqing Guangdong 526238 P. R. China; ^3^ ARC Centre of Excellence in Exciton Science School of Chemistry University of Melbourne Parkville VIC 3010 Australia; ^4^ Guangdong Provincial Key Laboratory of Nanophotonic Functional Materials and Devices School of Information and Optoelectronic Science and Engineering South China Normal University Guangzhou 510006 P. R. China

**Keywords:** colloidal assembly, dielectrophoresis, displays, electric field, water‐in‐oil droplet

## Abstract

Colloidal assembly is a key strategy in nature and artificial device. Hereby, an electromicrofluidic assembly platform (eMAP) is proposed and validated to achieve 3D colloidal assembly and manipulation within water droplets. The water‐in‐oil emulsion droplets autoposition in the eMAP driven by dielectrophoresis, where the (di)electrowetting effect induces droplet deformation, facilitating quadratic growth of the electric field in water droplet to achieve “far‐field” dielectrophoretic colloidal assembly. Reconfigurable 3D colloidal configurations are observed and dynamically programmed via applied electric fields, colloidal properties, and droplet size. Binary and ternary colloidal assemblies in one droplet allow designable chemical and physical anisotropies for functional materials and devices. Integration of eMAP in high throughput enables mass production of functional microcapsules, and programmable optoelectronic units for display devices. This eMAP is a valuable reference for expanding fundamental and practical exploration of colloidal systems.

## Graphical abstract

3D colloidal assembly in water droplet is achieved via dielectrophoretic induced droplet positioning, (di)electrowetting induced droplet deformation, and dielectrophoretic induced particle assembly, obtaining multiple tunable assembly configurations for constructing anisotropic microcapsules with binary colloidal structure or switchable microvalves which enables applications in functional materials or display devices.

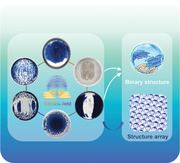

## 1 Introduction

Colloidal and molecular self‐assembly are key strategies for creating functional materials in biological, chemical, and physical systems.^[^ ^1^ ^]^ Active control over colloid particles has been intensively studied owing to its ability to mimic natural structures^[^ ^2^ ^]^ and because it offers a pathway to dynamically build and control material function.^[^ ^3^ ^]^ It is thus an attractive approach for the design of dynamically tunable structures from colloidal building blocks via active colloidal assembly.

Many efforts have been devoted to the formation and application of diverse complex structures at solid surfaces.^[^ ^1^, ^4^ ^]^ Liquid interfaces have also been used as templates to form colloidal assemblies.^[^ ^5^ ^]^ At the oil–oil interface, clay ribbons have been formed via electrohydrodynamic (EHD) forces in a uniform electric field.^[^ ^6^ ^]^ Janus magnetic particle assembly at oil–water interfaces interacting with the magnetic field has also been investigated.^[^ ^7^ ^]^ Despite all these recent advances in colloidal assembly, e.g., 2D arrays^[^ ^4f^ ^]^ or 3D clusters,^[^ ^8^ ^]^ the assembly mechanisms dependent on solid or liquid interfaces limit the formation of complex structures. Strategies capable of transporting colloidal assemblies to different spatial heights and thus flexibly tuning their 3D assembly structures,^[^ ^9^ ^]^ are still highly desired.

Liquid droplets provide confined spherical spaces ranging from nanometer to millimeter length scales; they also offer tunable shapes and smooth interfaces within the molecular range. Various strategies have been employed for producing diverse colloidal assemblies within a droplet or at the droplet interface.^[^ ^10^ ^]^ Magnetic assembly within a droplet has been exploited to enhance achievable functionalities, such as tunable droplet shape^[^ ^11^ ^]^ or colloidal crystal displays.^[^ ^12^, ^13^ ^]^ Compared with magnetic fields, electric fields are generally applicable to particles with a greater variety of compositions and offers richer manipulation modes;^[^ ^14^ ^]^ thus, they have been widely applied to control colloidal assembly,^[^ ^15^ ^]^ cell enrichment,^[^ ^16^ ^]^ photonic crystal structure,^[^ ^17^ ^]^ and particle motion.^[^ ^18^ ^]^ Nevertheless, electrically driven colloidal assembly within liquid droplets has rarely been studied, even though particle assembly at liquid–liquid interfaces or within oil droplets has been investigated.^[^ ^19^ ^]^ Furthermore, water‐in‐oil (W/O) emulsions are extensively used to create spherical water spaces and have gained widespread interest in the fields of colloidal assembly,^[^ ^20^ ^]^ biological research,^[^ ^21^ ^]^ and food engineering.^[^ ^22^ ^]^ Electric field‐driven colloidal assembly within confined micro‐spaces offers opportunities for both fundamental research and practical applications. However, existing approaches still face challenges of controllability, complexity, or integrability.

One issue is that most of the electric field is distributed within the oil phase rather than in the water phase^[^ ^23^ ^]^ because of the dielectric difference between oil and water. Increasing the amplitude of alternate current (AC) voltage can solve this problem, however this introduces the EHD effect.^[^ ^24^ ^]^ Unlike the 2D colloidal structure at the oil–oil droplet interface formed based on EHD,^[^ ^6a^ ^]^ in a water droplet suspended in an oil medium, the EHD effect will disrupt the orientational order of a 3D colloidal structure, diminishing the structural diversity and stability. This has impeded wider investigation and potential applications.^[^ ^25^ ^]^ Electrowetting has been applied to manipulate droplets; however, the commonly used strategy to maximize droplet wettability is unfavorable for introducing electric fields into water droplets,^[^ ^26^ ^]^ therefore hindering the manipulation of colloidal assemblies,^[^ ^27^ ^]^ leaving the intrinsic link between droplet wetting and in‐droplet colloidal assembly still unknown.

Here, we report on an electro‐microfluidic assembly platform (eMAP) to construct and manipulate 3D colloidal assembly within water droplets based on dielectrophoresis induced droplet positioning, (di)electrowetting induced droplet deformation, and dielectrophoretic colloidal assembly. Water‐in‐oil emulsions are spread onto an eMAP surface covering multiple electrode pairs. Upon application of an alternating current (AC) voltage between the pairs of electrodes, the water droplet can automatically position itself within the gaps of the electrode array and the droplet may then slightly deform in response to dielectrophoresis and (di)electrowetting. Such deformation behavior facilitates quadratic growth of the electric field within the water droplet, ensuring dielectrophoretic colloidal assembly can occur while avoiding strong EHD effects. The quasi‐spherical droplet causes physical confinement of colloids and strong bending of internal electric field lines; these are used to achieve manipulation of colloid motion and active colloid assembly. Using this method, dielectrophoretic assembly and manipulation of particles can be conducted at much higher elevations above the electrode than current methods,^[^ ^28^ ^]^ thereby enabling a greater range of structures to be investigated. Various superstructures within water droplets have been observed and harvested by tuning the inner fluidic and electric fields as well as the colloid properties. Such a controllable strategy is further demonstrated and extended to the formation of anisotropic microgels from binary colloidal assembly within a curable droplet, while controllable, electrically driven optofluidic valves and display devices based on high throughput eMAP integration are also presented.

## 2 Results

### 2.1 Electro‐Microfluidic Assembly Platform

**Figure 1:**
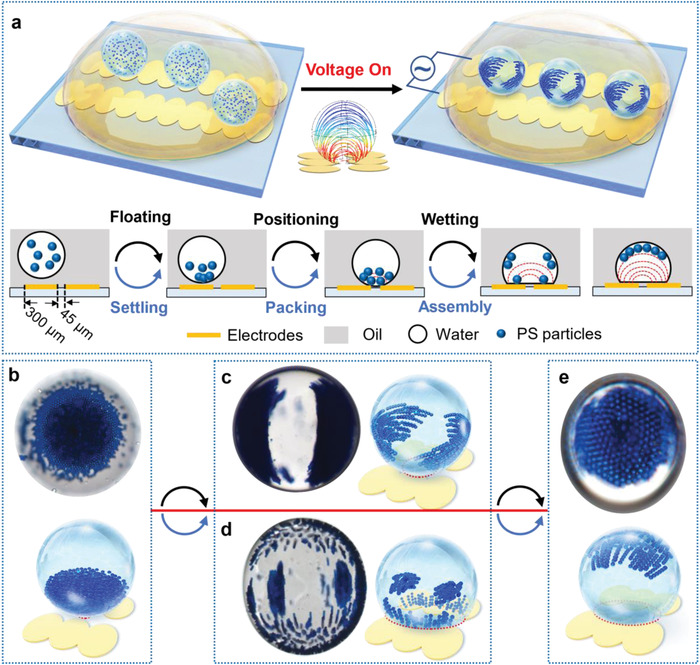
Schematic and illustration of eMAP. a) Schematic of the eMAP for manipulating water droplets and the particles inside the droplet. With the application of an AC electric field via the electrodes, the freely floating water droplets are captured onto the patterned electrode arrays. and the smaller internal particles settle and pack at the bottom. Subsequently, (di)electrowetting induced droplet deformation occurs and induces particles to assemble into various configurations accordingly. b–e) Top‐view optical images and drawings of representative colloidal structures achieved by varying *U* and *f*. b) the initial‐state (*U* = 0) with the colloid particles densely packed at the bottom of the water droplet, c) the spreading‐wing‐like structure, d) the rising chain‐like structure, and e) the top cap‐like structure.

**Figure** **1**a shows the conceptual diagram of the electro‐microfluidic assembly platform (eMAP), which consists of a multiphase microfluidic system on a fluoropolymer‐coated electrode pattern. The water droplets (hundreds of microns in diameter) comprising well‐dispersed colloidal particles are suspended in a continuous phase (typically an oil phase) with low permittivity. Silicone oil is selected as the oil phase because of its low volatility. This helps to avoid solvent evaporation, which can induce flow fields at the oil–water interface, thereby ensuring stable colloidal assemblies can be formed. Colloid particles with hydrophilic surfaces are used to ensure high dispersibility in the water droplets. The multiphase emulsion spreads on the Hyflon (≈900 nm) coated caterpillar electrode array with the water droplets initially randomly distributed within the oil (continuous phase). When an electric field is applied, several events occur. First, there is autopositioning of the water droplet, driven by dielectrophoresis, to the region between electrode pairs where the highest electric field strength is, due to the higher permittivity and conductivity of the water compared to the oil. A second event is droplet deformation due to the (di)electrowetting effect. Finally, there is the motion and 3D assembly of colloidal particles within the water droplet driven by dielectrophoresis facilitated by the deformation enhanced internal electric field.

Returning to the illustration in Figure 1a, we note that the water droplets settle downward in the oil due to their density difference, touching the oil‐lubricated hydrophobic surface with their positions and contact areas on the electrode pattern controlled by the applied electric field. Considering the droplet size and the need to avoid high voltage‐induced electrolysis, the caterpillar electrodes are organized in a row with a pitch of 345 µm and paired with a gap of 45 µm. Figure S1 (Supporting Information) presents the optimized structure and relevant electrical features of the eMAP. The short axes of the elliptical electrodes are 160 µm long with a 20 µm overlap with each other. By applying an alternating current (AC) electric field, water droplets automatically migrate to the regions between the two rows of electrodes and experience wetting behavior due to dielectrophoretic (DEP) and electrowetting (EW)/dielectrowetting (DEW) effects, respectively. Some water droplets may fuse with each other in the vicinity of the gap due to the electrofusion effect, which can be avoided by reducing water droplet concentration, slowing down the fluid flow, and lowering the initial AC field strength.

The colloid particles typically retain their dispersibility and remain randomly distributed in the water droplet for about 30 s. The particles then begin to assemble into curved dipolar chains along the electric field lines, being somewhat similar in appearance to a mitotic spindle during cell division (Figure S2, Supporting Information). Due to their density difference, the majority of particles settle down to the floor of the droplet after a while, forming a densely packed state, namely the initial state, as depicted in Figure 1b. For instance, polystyrene (PS) particles (particle diameter, *d* = 7 µm) settle to the bottom of a water droplet (*D* ≈ 350 µm) within 120 s. Such an initial state can be recovered after turning off the electric field, however the particles cannot return to the random distribution state after activation. When starting from this initial state, various reconfigurable 3D colloidalstructures are observed by tuning the voltage amplitude (*U*) and frequency (*f*) of the applied electric field, as shown in Figure 1c–e, namely the spreading‐wing‐like, rising‐chain‐like, and top‐cap‐like configurations. These colloidal assembly structures are reversible and programmable with all states being either directly or indirectly switchable.

### 2.2 Droplet Positioning and Deformation

**Figure 2:**
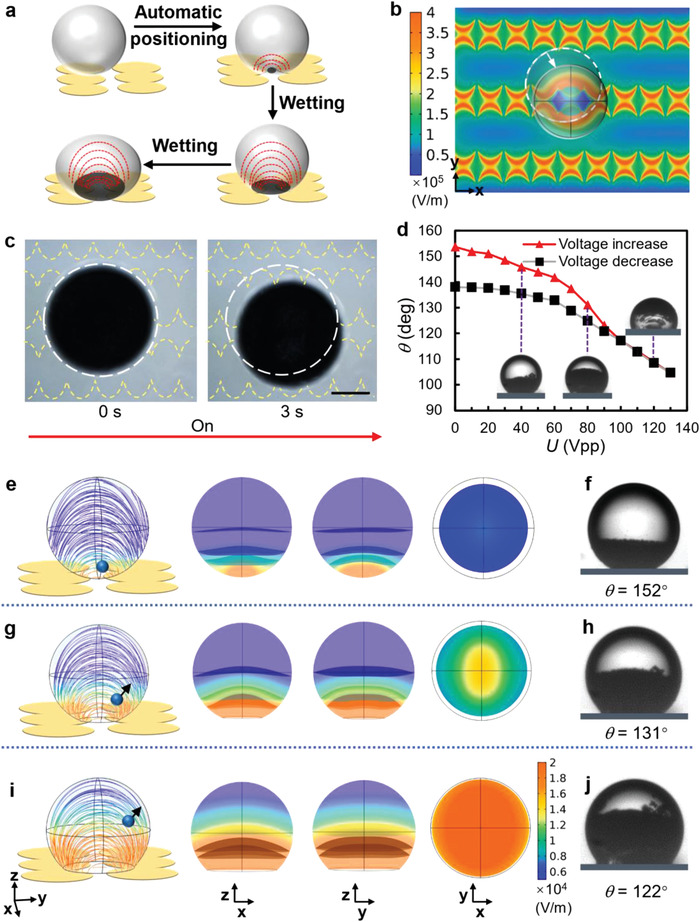
Dielectrophoresis induced droplet positioning and electrowetting/dielectrowetting induced droplet deformation on eMAP. a) Schematic illustration showing the automatic positioning and wetting of awater droplet on the eMAP surface. The red dashed lines indicate the inner electric field and the gray circle areas mark the contact area between the droplet and the substrate. b) Simulated electric field intensity mapping of the electrode array at 60 V_pp_ and 300 kHz. c) Experimental observation of automatic positioning of a droplet at an AC electric field of 60 V_pp_ and 300 kHz. Scale bar, 200 µm. d) Droplet contact angle (*θ*) changing with the applied voltage (*U*) according to the DEW effect at *f* = 300 kHz. e–j) Variation of the internal electric field distribution as a function of *θ* of 152° e,f), 131° g, h) and 122° i,j) with the applied electric field fixed at 60 V_pp_ and 300 kHz. Results in the *x*‐*y* section represent the electric field distribution at *z* = 100 µm (height) from the electrode surface. The droplet diameter is 380 µm in d–j).

Since the dielectric properties (permittivity and conductivity) of water droplet are much higher than that of the oil medium and the hydrophobic insulator, the electric potential mainly dissipates across the low polarity phase (oil and insulator) at low frequency. As a result, only weak electric field strength is generated within the water droplet, which is unfavorable for colloidal assembly. Increasing *U* at low frequency can increase the field strength, but is accompanied with the generation of strong EHD effects which interferes the assembly performance. Therefore, high‐frequency AC electric field is employed in eMAP in this work. Moreover, under a proper AC electric field, the water droplet undergoes two processes: the automatic positioning onto the gap of the caterpillar electrode arrays and the wetting on the hydrophobic insulator surface, essentially induced by the DEP and DEW effects, respectively, as shown in **Figure** **2**a. A water droplet is subjected to a positive dielectrophoretic (pDEP) force which causes the droplet to migrate to the gap area among electrodes (typically within 3 s) (Figure 2b,c), and the DEW effect induces the decrease of the contact angle (*θ*) with the increase of *U*, as shown in Figure 2d. As a result, the electric field strength inside the water droplet (*E*_in_) significantly increases due to the high magnitude of the fringing field adjacent to the electrode edge. Benefiting from the DEP and DEW effects, a high *E*_in_ is achieved in a deformed water droplet in the eMAP. This sequential and synergetic effect is the key to programmed colloidal assembly within water droplet.

The mean value of the DEP force can be calculated from 〈**F**_DEP_〉 = 2*πε*_m_Re[*f*_CM_]*R* ^3^∇|**E**_rms_|^2^, where **E**_rms_ is the mean square root (RMS) electric field. For a sine wave, the RMS value is ≈0.707 times the peak value. ∇|**E**_rms_|^2^ represents the gradient of the square of **E**_rms_, *ε*_m_ is the permittivity of the surrounding medium, *R* is the particle radius, and Re[*f*_CM_] represents the real part of *f*_CM_. The parameter *f*_CM_ is the Clausius‐Mossotti (CM) factor which represents the effective polarizability described by $f_{\mathrm{CM}}\;=\;(\frac{{\tilde{\varepsilon }}_{p}-{\tilde{\varepsilon }}_{m}}{{\tilde{\varepsilon }}_{p}+2{\tilde{\varepsilon }}_{m}})$, where ${\tilde{\varepsilon }}_{p}$ and ${\tilde{\varepsilon }}_{m}$ indicate the complex permittivity of the particle and the surrounding medium, respectively. The complex permittivity responses to the angular frequency (*ω* = 2*πf*) of the applied AC electric field and can be written as $\tilde{\varepsilon }\;=\;\varepsilon -j(\frac{\sigma }{\omega })$. When a particle is more polarizable than the surrounding medium (Re[*f*_CM_] > 0), it will be attracted towards the high *E* region due to pDEP, whereas it will be repelled towards the low *E* region in the case of negative dielectrophoresis (nDEP). Therefore, the colloid particles with lower polarizability than water will experience nDEP (e.g., PS particle), moving from the floor toward the top of the water droplet, whereas a particle with higher polarizability experiences a pDEP force (e.g., metal coated particles) being attracted to the floor of the droplet. In this eMAP, the water droplet is also more polarizable (Re[*f*_CM_] > 0) than the surrounding oil medium, and thus experiences pDEP forces. However, since the droplet diameter is greater than the scale of the electric field nonuniformity, the calculation of the exact *F*_DEP_ value of the water droplet must take account of multipole force terms.^[^ ^29^ ^]^

The conductive water droplet is surrounded by insulating silicone oil and Hyflon, which shield the electric field from entering the water droplet for low‐frequency AC or DC electric fields. The relaxation frequency (*f*_relaxation_) where the dielectric polarization takes over from the conduction current regime,^[^ ^30^ ^]^ is therefore calculated as *f*_relaxation_ = *σ* (2*πε*)^−1^, where *σ* and *ε* are the conductivity and permittivity of the droplet, respectively. The conductivity of deionized water gradually increases from 0.0055 to ≈0.2 mS m^−1^ when exposed to air due to the absorption of CO_2_;^[^ ^31^ ^]^ thus, *f*_relaxation_ is ≈45 kHz. The frequencies higher or lower than this value are defined as “high frequency” and “low frequency,” respectively. When *f* > *f*_relaxation,_ the orientational polarization from water molecules dominates, because the time is too short to form an electrical double layer on the droplet surface ‐ a process that is limited by the slow ion diffusion. Hence, the water droplet can be regarded as a perfect dielectric sphere with a relative permittivity of ≈80, which can no longer shield the applied electric field. Meanwhile, a water droplet is generally affected by the dielectrowetting effect at such a high frequency,^[^ ^32^ ^]^ and consequently there is a decrease in contact angle (*θ*) and an increase in the contact area between the droplet and the substrate surface. Correspondingly, the droplet's height (*h*) reduces in the z‐direction (Figure 2d). The relationship between *h* and *U* is given by $h^{2}\left(U\right)\;=\;h_{0}^{2}-\frac{\varepsilon_{w}-\varepsilon_{o}}{4\delta \gamma_{\mathrm{ow}}}U^{2}A$, where *h*_0_ is the equilibrium height of the water droplet relative to the height at zero voltage, *δ* is the penetration depth, and *A* is the cross‐sectional area of the droplet which is calculated as *A* = *hl* with *l* the droplet's size in the *x*‐axis direction. *U* decays from the substrate surface into the liquid to a depth of *δ*, which is given by,^[^ ^33^ ^]^ U(z)=U·exp(−2z/δ), where *δ* is calculated to be ≈42.9 µm for a water droplet in this eMAP. To verify the impact of the DEW effect, the internal electric field distribution as a function of the degree of wetting (denoted by contact angle) is simulated using COMSOL 5.5, and the results are shown in Figure 2e–h.

For a spherical droplet, the contact area can be set to ≈0, and the droplet is positioned at the gap of electrode pairs and the droplet's bottom surface is tangential to the substrate surface (Figure 2e,f), while for a droplet driven by DEW, the contact area obtained in experiments is used as the reference in the simulation (Figure 2g,h). In contrast to a perfectly spherical droplet, the isosurface of the electric field in a deformed droplet is much more bent in the *x*–*z* plane, while the high intensity electric field area (red) in the *x*–*z* plane is larger, indicating that a more deformed droplet can induce a stronger *E*_in_ and a correspondingly higher bending of the nonuniform field distribution. The top view of the electric field distribution in the equatorial plane (*x*–*y* plane) supports this conclusion. Figure 2i,j shows the simulated and experimental results at weak wetting status with *θ* = 122°. Figure 2f,h,j demonstrates that the height of the PS particle (7 µm, 8 wt%) assemblies in the deformed droplet rises with increased wetting (denoted by the decrease in *θ*), indicating there is an increase in the magnitude of *E*_in_ at the same *U* and *f*. In practice, an instantaneous change in *θ* for a droplet at the same *U* and *f* values can be experimentally achieved using the *θ* hysteresis effect via quick “low‐high” voltage alternating cycles. A larger contact angle change will induce a higher value of *E*_in_ for the same value of *U*. In all experiments except Figure 2g,h,j, the contact angle change/hysteresis is set to ≈15°, corresponding to voltage cycles in the range 0–130 V_pp_.

At low frequency (*f* < 45 kHz), the ion motion in water causes an Ohmic current and an electric double layer forms on the surface of the water droplet. The electric double layer (Debye length, ≈90 nm) can be considered as a parallel capacitor in series with a dielectric layer that shields the electric field from entering the water droplet. Therefore, an increase in *E*_in_ results in the formation of different particle distributions (Figure S3a,b, Supporting Information). Meanwhile, at low frequency, the electrowetting effect induces an increase in contact area of the water droplet on the eMAP (Figure S3c, Supporting Information). The electric field distribution near the base of the water droplet tends to match the electrode shape as well (Figure S3b, Supporting Information).

### 2.3 Programmable 3D Colloidal Assemblies in Water Droplets

The 3D superstructures of colloidal assemblies in a droplet are mainly determined by the internal electric field distribution and the colloidal properties. As presented above, at the same *U* and *f*, the strength and bending degree of the internal electric field in a water droplet are strongly affected by the droplet position and its degree of deformation (wetting effect). Insulating PS particles with low conductivity and low relative permittivity (*ε*_p_ ≈ 2.5) exhibit a lower polarizability (Re[*f*_CM_] < 0) than water (*σ*_m_, ≈ 0.2 mS m^−1^, *ε*_m_, ≈80), and hence experience nDEP forces when a high‐frequency AC electric field is applied. Therefore, PS particles move from the floor (high *E*_in_ region) towards the top (low *E*_in_ region) of a water droplet.

#### 2.3.1 Wing‐Like Structures

**Figure 3:**
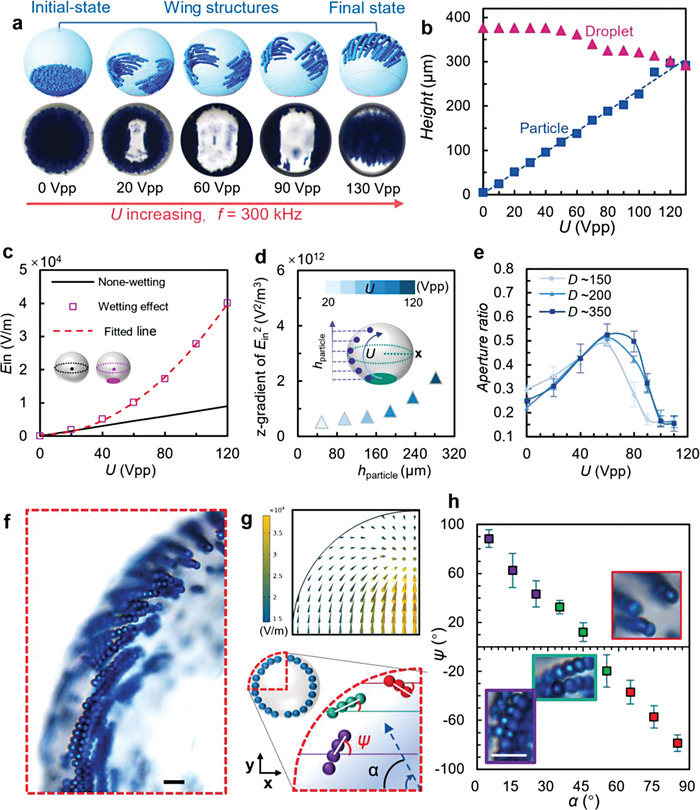
Manipulation of wing‐like and cap‐like colloidal assemblies in droplets. a) Structure evolution with *U* at a fixed frequency, *f* = 300 kHz. The upper row shows 3D schematics of the putative assembly position. The droplet (*D* = 274 µm) contains 8 wt% PS particles. b) Scatter plot showing the height of the particle clusters (blue) and droplets (magenta) as a function of *U*. The droplet (*D* = 376 µm) contains 1 wt% PS particles. c) Simulation results for the inner electric field strength (*E*_in_) as a function of *U. E*_in_ grows linearly and quadratically with *U* for a droplet under nonwetting and wetting effects, respectively. The wetting degrees used for the simulations refer to the experimental results. The coordinate of the simulation center point is at (*x*, *y* = 0, *z* = 140 µm). d) *z*‐gradient of *E*_in_^2^ distribution near the droplet interface corresponding to the simultaneous linear increase in *U* and *h*_particle_. e) Aperture ratio (opening area/total droplet area) as a function of *U* for droplets with different diameters. f) Optical image of the particle assemblies of a wing structure in a 689 µm water droplet at *U* = 60 V_pp_ and *f* = 300 kHz. Scale bar, 20 µm. g) Simulated electric field distribution (upper) and schematic illustration of a quarter of the droplet defining *Ψ* and *α*. (lower). h) Plot of *Ψ* as a function of *α*, expressing the feathery‐like structures with a spreading‐wing‐like morphology. Scale bar, 20 µm. The data are obtained by analyzing the top view images, which may be slightly affected by the droplet's refraction.

In the eMAP, the colloidal assembly performance can be well controlled and dynamically programmed via the applied AC electric fields. When an AC electric field of *U* = 60 V_pp_ and *f* = 300 kHz is applied, a mushroom‐like electric field distribution is formed inside the droplet with *E*_in_ weakening from the center to the edge in the *x*–*y* plane and from the bottom to the top along the *z*‐axis (Figure 2g). As a result, the PS particles levitate and spread rapidly toward the water interface (in the *x*‐axis) where *E*_in_ is relatively low, forming particle chains near the equator, balanced by both the DEP force and gravity. The clusters of the particle chains in the droplet show a “spreading‐wing” like 3D structure viewed from the top (**Figure** **3**a and Movie S1, Supporting Information); henceforth, we simply call this a “wing‐like structure.” The majority of PS particles preferentially remain within the droplets because of its wetting preference and image charge effects.^[^ ^34^ ^]^ The presence of a large particle‐free region at the droplet interface also indicates that the PS particles tend to stay in the water phase, and the solids content of 8 wt% is theoretically sufficient to allow close packing of PS particles (*d* = 7 µm) over the entire droplet interface (*D* < 310 µm). The particle motion can be categorized into two types: the major proportion of the particles are distributed inside the droplet and form particle chains, while a very small proportion (less than 0.5 wt%) of particles are trapped at the oil–water interface possibly as a result of their surface defects,^[^ ^35^ ^]^ sliding slowly upward along the droplet interface. The wing‐like structure is typically formed within 2 s of applying an AC electric field.

The water droplets containing 1.0 wt% blue PS particles are used to clearly show the position and dynamic assembly of the colloids in the eMAP (Figure 3b,f–h). Figure 3b shows the linear growth of the height of the particle‐assembly (*h*_particle,_ the highest point of the particle assemblies in the droplet) and *U* in a droplet (*D* = 367 µm). *E*_in_ exhibits quadratic growth (R‐squared = 0.999) with *U* facilitated by the wetting effect, rather than the well‐known linear relationship without the wetting effect, as shown in Figure 3c. Considering *E*_in_ at a certain point is inversely proportional to the distance to the electrode center gap (*r*_in_) within the droplet,^[^ ^30^ ^]^ this can be expressed as *E*_in_ ∝ *r*_in_^−1^. Since wing‐like structures are commonly formed along the droplet inner interface, *r*_in_ can be simply calculated as *r*_in_ = (*Dh*_particle_)^1/2^_._ As a result, we derive the relationship among *U*, *D, h*_particles_ and the electric field strength distribution at a wing structure as *E*_in_ ∝ *U* ^2^(*Dh*_particle_)^−1/2^.

To understand the causes of the linear relationship between *U* and *h*_particles_, the *z*‐axis component of *E*_in_ corresponding to *U* and *h*_particles_ is investigated. Figure S4a (Supporting Information) and Figure 3d present the *z*‐component of *E*_in_ and *z*‐gradient of the *E*_in_^2^ distribution as functions of *U* and *h*_particle_. The comparable values demonstrate that the *z*‐axis component of *E*_in_ is independent of the simultaneous linear increase in *U* and *h*_particle_, which implies that there is no major variation in particle polarization along the *z*‐axis. Furthermore, when *U* increases from 0 to 80 V_pp_, the particles migrate higher but remain in the southern hemisphere (*h*_particle_ < 180 µm), while the *z*‐gradient of *E*_in_^2^ only grows to 1.3 times its original value. This suggests the z‐axis component of the dielectric force (*F*_DEP_ ∝ ∇*E*_in_^2^) acting on the particles is almost constant when *h*_particle_ and *U* are linearly related. However, there is a ≈1.3 times increase in the *z*‐gradient of *E*_in_^2^ when the particles move from the equator toward the droplet ceiling in the northern hemisphere. This means that the particles are constrained by the droplet top interface and thus require higher DEP forces to hold them at the equilibrium position.

In addition, we find that the evolution of the wing‐like structures (reflected by the aperture ratio) is influenced by the droplet size. As shown in Figure 3e, for the three droplets with different sizes, the aperture ratio changes with *U* in the same way. The similar positive slopes corresponding to the three droplets imply that the lifting rule for the colloids is adaptive. One piece of evidence is that, even within droplets which differ in size by a factor of two, all wing‐like structures exhibit a maximum aperture ratio at 60 V_pp_, corresponding to the particles rising to the droplets’ equators. This feature may also relate to the wetting effect which can change *E*_in_. With a further increase in *U*, the aperture ratio decreases as the two wings levitate to the top and eventually merge to form a top‐cap‐like structure (final state in Figure 3a). During this stage, the decrease in the aperture ratio is because of the growth of *h*_particle_ due to the curved confinement of the droplet. Therefore, the particles in smaller droplets are easier to reach the top interface, as illustrated in Figure 3e, and the colloidal structures in the smallest droplet (≈150 µm) can reach the ceiling at the lowest value of *U* ≈ 90 V_pp_. Under strong nDEP forces, the particle chains of the wing‐like structure are squeezed to eventually form top‐cap‐like structures (hexagonally close‐packed, crystalline assemblies) within ≈10 s (Figure S4, Supporting Information). The electrothermal effect that appears at high values of *U* (>130 V_pp_) may slightly change the particle chain orientation, and the wing‐like structures are still maintained. In addition, the observable droplet deformation is due to the nonuniformly distributed electric stress on the droplet surface^[^ ^36^ ^]^ and the DEW effect. As shown in Figure S4b (Supporting Information), a smaller droplet actuated at a higher *U* achieves a larger aspect ratio. As a result, by using monodisperse droplets, uniform wing structures can be obtained under the same AC field.

It is worth noting that there are highly ordered chains grown in the wing‐like structure, which is not achievable by traditional substrate‐based assembly methods. As shown in Figure 3f, the colloidal chains arrange themselves into feather‐like structures due to the competition among the chain–chain interactions, the electric stresses due to the nonuniform electric field, and the curved interfacial confinement of the droplet. Figure 3g depicts the simulated electric field distribution. The direction of the arrows in the blue region coincides with the particle chain orientation, confirming that the spatially distributed electric field lines can control the order of the assemblies. We denote the angle between the *x*‐axis and the colloidal chain as *Ψ* (Figure 3g) and this in turn varies with the circumferential angle *α* (Figure 3h). The values of *Ψ* for the purple, green, and red colloidal chains are >+35°, ‐35° to +35°, and <‐35°, respectively. As the radial direction changes from the *x*‐axis towards the *y*‐axis (i.e., *α* changes from 0 to 90°), *Ψ* varies correspondingly from about 88° to ‐78°. This means that the feather‐like structure is highly regular with the orientation varying over a wide range. Orthogonal sideviews of the assembled structures and the prepared large‐area structures are presented in Figure S5 (Supporting Information).

As mentioned above, *h*_particle_ is proportional to *U*, and *Ψ* coincides well with the electric field direction, demonstrating the possibility of forming stable and controllable assemblies within the deformed water droplet. To validate the scope of the wing‐like structure formation, the droplets with diameters in the range of 80–950 µm have been verified experimentally. As shown in Figure S6 (Supporting Information), starting from the initial state, wing‐like structures with various sizes, heights, and densities have been observed and programmed by varying *U* in the range of 20–140 V_pp_ and *f* in the range of 50–500 kHz.

#### 2.3.2 Rising Chain‐Like Structures

**Figure 4:**
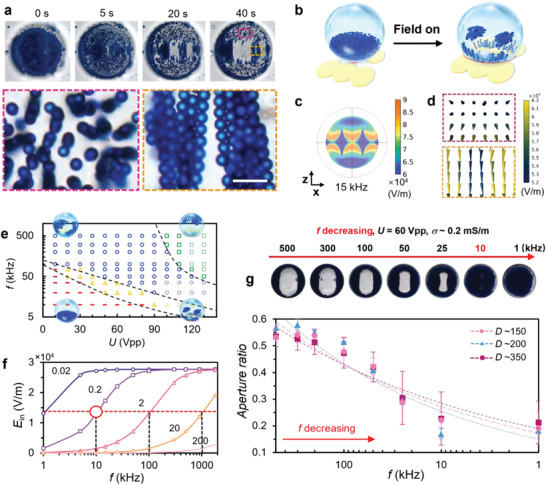
Evolution of the rising‐chain‐like colloidal assemblies at low frequency. a) Optical images taken during the assembly process showing differential behaviors in the areas above the electrode and between two electrodes in the same droplet (*D* = 476 µm). Insets are the magnified images showing the arranged colloidal chains vertical and parallel to the substrate surface. Scale bar, 20 µm. b) Schematic illustration showing the putative rising‐chain‐like structure based on experimental observation at *U* = 60 V_pp_ and *f* = 15 kHz. c) Simulated electric field map showing the distinctive field distribution corresponding to the electrode gap, the electrode center and the electrode edge areas at a height of 5 µm (*z*‐direction). d) Simulated local electric field lines over the *x*–*y* planes at z = 20 µm corresponding to the blue line marked area in b. e) Phase diagram showing regions of various colloidal structures formed at corresponding values of *f* and *U*. Dashed markers represent the theoretical predictions. f) Simulated electric field distribution at a fixed central point (*x* = *y* = 0, *z* = 100 µm) within the droplet as a function of f for a range of droplet conductivities (0.02, 0.2, 2, 20, 200 mS m^−1^) at *U* = 60 V_pp_. g) Images showing the evolution of the colloidal assemblies in droplets with various diameters as a functio of f and  the corresponding aperture ratios as a function of *f*. The concentration of the blue PS particles (*d* = 7 µm) is 8 wt%.

As described above, *f* influences the droplet wetting performance and the particle polarization. In this eMAP, at relatively low *f* (e.g., 15 kHz), the particle assembly in the water droplet is highly sensitive to the geometrical parameters, e.g., the shape, the size and the gap of the electrodes accompanied by the contact area between the droplet and the hydrophobic surface (Movie S2, Supporting Information). As shown in **Figure** **4**a,b, inside the droplet, the polarized particles can “grow” vertically (with a slightly tilted angle) above the electrode area and form “bridging” chains across the gap between two electrodes. The magnitude of *E*_in_ between the paired electrodes (diamond shape, blue) and in the centre of the electrodes (crescent shape, blue) is weaker than at the edges of the electrodes (orange and yellow), as shown in Figure 4c. Together, the *E*_in_ gradient (blue area) and the field line directions determine the position and orientation of the colloidal assemblies (Figure 4d). Inside the droplet, vertical chains growing along the electrical field lines are observed above the electrode region, with the chain length observed to be 2*d* to 4*d* (*d* = 7 µm), while the chain‐chain distance lies between *d* and 4*d*. This suggests a repulsive interaction between the vertically grown chains.

For the formation of “bridging” chains, it is observed that the particles first form chains along the electric field lines at the bottom interface of the droplet; afterward, the bridge's left and right sides rise more quickly than the center and gradually converge towards the center (Figure S7, Supporting Information). The “bridging” chains are fully assembled at ≈20 s corresponding to the lowest width of the “bridging” chains (*W*_bridge_), and then levitate at a *z* value of about 4*d* to 5*d* for a long period. The convergence of “bridging” chains is attributed to the lower value of *E*_in_ at the center (blue area in Figure 4d), while the chain lifting effect is determined by *E*_in_ ∝ *r*_in_^−1^, as mentioned above. Combining the rising and converging effects, the stable levitation of “bridging” chains can be achieved. Moreover, the “bridging” chains show attractive rather than repulsive chain‐chain interactions in this case. The long “bridging” chains (5*d* to 12*d* long) and their attractive interactions suggest there may be intrinsic correlations, i.e. longer range ordering forces (Figure S8, Supporting Information).

We further draw a phase diagram (Figure 4e) to summarize the colloidal structures observed for specific values of the relevant variables (*U* and *f*), and simulate *E*_in_ versus *f* for different droplet conductivities (Figure 4f). These results help clarify the criteria and span of *f*, *U*, and the droplet conductivity, and aid our understanding of the formation mechanism of the colloidal structures in droplets. As presented in Figure 4e,f, an increase in *f* and *U* induces an increase of *E*_in_, leading to a gradual lifting of the colloidal structure from the bottom to the equator, and then to the top of the droplet (corresponding to the red, blue, and green regions). For the droplet with *σ* ≈ 0.2 mS m^−1^, when *f* is close to *f*_relaxation_ (45 kHz), the value of *E*_in_ increases strongly with *f*, and reaches a plateau when *f* >>*f*_relaxation_, which is consistent with the gradually narrowing red region and the progressively widening blue and green regions in the phase diagram. The rising‐chain‐like structures (yellow region) can only form in a narrow range of values for *f* and *U*. This is to fulfill the requirement of a low *f* value for electrowetting and also a low value of *E*_in_ (< 2.5×10^4^ V m^‐1^) to maintain the assembly position away from the droplet's equator. Accordingly, a value of *f* >45 kHz satisfies the requirements to achieve a high value of *E*_in_ for the formation of wing‐like structures while avoiding strong electrothermal flow at high *U*;^[^ ^30^ ^]^ conversely, a value of *f* in the range of ≈10–45 kHz is suggested for the formation of the rising‐chain like structures.

For the droplet with a low conductivity of 0.02 mS m^−1^, the minimum *f* range expands to ≈1 kHz; whereas, when the droplet conductivity increases to 0.2 and then to 20 mS m^−1^, the minimum *f* required for DEP‐induced colloidal assembly shifts to 10 kHz and 1000 kHz. Thereby, for properly constructing the rising chain‐like structures in the water droplet, a liquid conductivity lower than 20 mS m^−1^ is highly preferred and achievable under mild conditions. Figure 4g demonstrates that the aperture ratio decreases with the logarithm of *f* for droplets with different diameters, which indeed corresponds to a reduction of *E*_in_. The similar slopes observed in Figure 4g also imply that the disassembly of the wing‐like structures is a synchronous response when decreasing *f*.

### 2.4 Universal eMAP for Various Colloids

Wing‐like structures can serve as intermediate states between the initial base state and the final top‐cap‐like structures programmed by the applied electric fields. An interesting question is whether a simple function of electric field can create spatial patterns in one droplet by using particles with various properties. To test this, different particles were mixed and dispersed in water to build structural and functional droplets, and **Table** **1** summarizes the particles used in the experiments.

**Table 1:** Information of the particles used in the experiments

Particle	Diameter [µm]	Solid content [wt%]
PS	≈4, 7, 10, 15, 20	8
SiO_2_	1, 3	5
Fe_3_O_4_@SiO_2_	≈2‐3	8
Cu	≈5	8
Silver‐coated hollow glass	≈5‐15	8
Yeast cell	≈7	8
Chlorite powder	≈10	8

**Figure 5:**
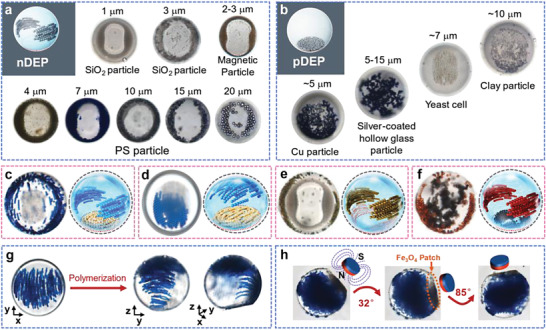
Universal eMAP for manipulating various colloids and constructing functional droplets/particles. a) Wing structures obtained from various particles driven by nDEP forces. b) Bottom packing structures obtained from different particles driven by pDEP. c) a 338 µm droplet with wing structure of 7 µm blue PS particles (6 wt%) and bottom packed yeast cells (6 wt%) and d) a 236 µm droplet with cap‐like structure of 7 µm blue PS particles (6 wt%) and bottom packed yeast cells (6 wt%), e) a 319 µm droplet with two wings of 1 µm silica particles (4 wt%) and 4 µm yellow PS particles (4 wt%) at different heights, f) a 314 µm droplet with a wing structure of 7 µm red PS particles (4 wt%) near the equator and bottom packed 5–15 µm silver‐coated, hollow glass particles (4 wt%) around the electrode shape. Optical images of crosslinked microgels obtained by UV exposure (365 nm, 600 mV for 3s) of g) a 254 µm droplet with 7 µm PS particles assembled at 80 V_pp_ and 300 kHz and h) a 366 µm droplet with 7 µm blue PS particle (6 wt%) and 2–3 µm Fe_3_O_4_@SiO_2_ magnetic particles (6 wt%) assembled at 60 V_pp_ and 200 kHz.

The permittivity and conductivity of SiO_2_ and PS particles are similar, and thus both particles experience nDEP forces and form wing‐like structures in the water droplets in the eMAP, as shown in **Figure** **5**a. SiO_2_ particles with a *d* of 3 µm and PS particles with *d* of 4, 7, 10, 15 and 20 µm can assemble into wing‐like structures at *U* = 60 V_pp_ and *f* = 300 kHz. When *d* is smaller than 5 µm, the particle's surface conductivity (*K*_s_) dominates,^[^ ^37^ ^]^ resulting in an increased apparent conductivity, which is given by, $\;\sigma_{p}\;=\;\sigma_{\mathrm{bulk}}+\frac{2K_{s}}{R}$, where *σ*_bulk_ is the bulk conductivity of the particles, which is negligible for nonconducting particles. Moreover, the particles with conductivity higher than water are subjected to pDEP forces when *f* < 270 kHz (Figure S9, Supporting Information). Thus, it is necessary to increase *f* to maintain nDEP for the particles with small sizes or high conductivity. For instance, 1 µm SiO_2_ particles and 2–3 µm Fe_3_O_4_@SiO_2_ magnetic particles present obvious wing‐like structures at *U* = 40 V_pp_ and *f* = 10 MHz, and *U* = 120 V_pp_ and *f* = 500 kHz.

Conductive particles were also used to demonstrate the feasibility of pDEP induced assembly in a water droplet. ≈5 µm Cu particles, 5–15 µm silver‐coated hollow glass particles, and ≈10 µm clay particles all exhibit higher polarizability than water droplets (*f*_CM_ > 0); therefore, they are subjected to pDEP forces and attracted to the bottom of the droplets at *U* = 60 V_pp_ and *f* = 300 kHz, as shown in Figure 5b. Unlike particle sedimentation, this field‐controlled assembly happens quickly. The different particle arrangements are a complex function of the particle dielectric properties, zeta potential, and surface wettability.

In addition, natural cells are representative of biocolloids that experience pDEP force in eMAP. As shown in Movie S3 (Supporting Information), the yeast cells in a water droplet assemble into cell chains, growing vertically from the bottom while migrating to the central area to form a flytrap‐like structure, when an electric field of 60 V_pp_ and 300 kHz is applied via the eMAP. We foresee that the capability to generate novel, in‐droplet cell arrangements can be used to build artificial biological patterns, for instance tissues or organs.

### 2.5 Construction of Anisotropic Microgels from Colloid Mixture in Droplets

Although diverse structures have been achieved by tuning *U* and *f*, more complex structures can also be created by differentiated assembly behaviors of various colloids in the same droplet. As shown in Figure 5c–f, two distinguishable structures from different particle assemblies are built in one water droplet with positions and architectures being programmed via the applied electric field in the eMAP. These colloidal structures can be photocured to form functional microgel particles via a polymerization process. The polymerizable droplets (Figure 5c–h) are prepared by using the water solution containing 20 wt% PEGDA (molecular weight of 575) and 1 wt% photoinitiator (2‐hydroxy‐2‐methylpropiophenone, 1173). The total particle concentration is optimized in the range of 8–12 wt% for clear observation of the dynamic processes and resultant structures.

Droplets consisting of biological and artificial particles at different locations are also obtained via the assembly of 6 wt% yeast cells and 6 wt% blue PS particles (*d* = 7 µm) as a result of the pDEP and nDEP forces, respectively (Figure 5c and Movie S4, Supporting Information). At *U* = 60 V_pp_ and *f* = 300 kHz, the biological cells are subjected to pDEP and thus tightly stacked at the bottom (occupying 6.3% surface area over the droplet), and the PS particles form wing structures near the equator. When increasing *U* to 80 V_pp_ at *f* = 300 kHz, the cell and PS particle assemblies migrate to the floor and ceiling of the droplet of prepolymer solution, respectively, as presented in Figure 5d.

Inorganic and organic polymer particles with different dielectric properties, similar dielectric properties and different densities, or similar dielectric properties and different sizes, can form wing‐like structures at different height levels within the same droplet. The droplets containing a binary particle suspension of 4 wt% SiO_2_ particles (*d* = 1 µm, nDEP) and 4 wt% yellow PS particles (*d* = 4 µm, nDEP) are driven to form two distinct wing structures in the southern hemisphere and near the equator at *U* = 40 V_pp_ and *f* = 10 MHz, respectively, as presented in Figure 5e. The top image shows a distinguishable gap between the two wing structures in yellow and grey, due to the differences in the dielectrophoretic forces (∝ *d* ^3^). The height difference results in different wing widths of ≈27 µm for the PS particles and ≈65 µm for the SiO_2_ particles. This suggests that particles with similar dielectric properties but different sizes or densities can be used to construct microgels with various patches using this eMAP.

To form droplets with conductive and insulating patches at controllable positions, metal and polymer particles are mixed and dispersed in the same water droplets. As shown in Figure 5f, when an electric field of 60 V_pp_ and 300 kHz is applied in the eMAP, an anisotropic droplet is formed with a red insulating patch comprising assembled 7 µm PS particles near the equator and a concomitant conductive patch comprising packed, silver‐coated, hollow glass particles with *d* = 5–15 µm at the bottom (occupying 10.4% of the droplet surface area).

As presented above, the addition of the prepolymer content in the droplet does not obviously affect the particle assembly behaviors in this eMAP. To demonstrate the possibility of preparing solid particles, the water droplet containing assembled chains of PS particles is polymerized by UV exposure. After crosslinking, an anisotropic microgel is obtained, as presented in Figure 5g, with the internal colloidal structure being well retained after turning off the electric field. Moreover, an anisotropic microgel with PS particles assembled near the equator and Fe_3_O_4_@SiO_2_ particles packed at the floor is also obtained by UV exposure, and this microgel can rotate in response to an applied magnetic field (Figure 5h). The magnetic field (≈125 mT) is provided by cylindrical neodymium iron boron magnets with a diameter of 15 mm and a thickness of 4.0 mm, facing the hydrogel at a ≈20 mm distance.

### 2.6 Active Electro‐Optical Performance and Applications

**Figure 6:**
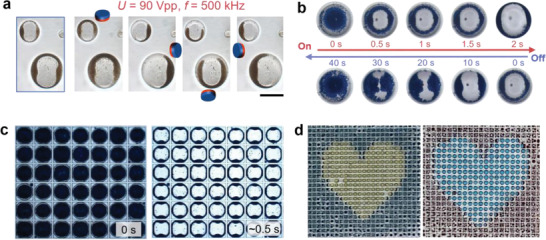
Optoelectronic properties and functions of the eMAP. a) Electric and magnetic dual‐field driven colloidal assembly in a water droplet. Scale bar, 200 µm. b) Dynamic configurations of colloidal assembly in a 350 µm water droplet actuated at *U* = 60 V_pp_ and *f* = 300 kHz. c) High throughput integration of eMAP for manipulating particle assembly in a large quantity of droplets (*D* ≈ 350 µm), showing mass production of particle assemblies and optical valves. d) Display performance of an integrated eMAP device created by using blue particles suspended in yellow water (left) and red particles in blue water (right). The particle concentrations are 8 wt% in a–c), and 12 wt% in d).

The proposed eMAP provides a powerful platform for studying stimuli‐responsive phenomena. The magnetic particles of Fe_3_O_4_@SiO_2_ with *d* of 2–3 µm have been dispersed in a water droplet to investigate their dual‐field responsive behavior in the eMAP. When an AC electric field of *U* = 120 V_pp_ and *f* = 500 kHz is applied, a wing structure is formed as shown in the blue box of **Figure** **6**a. Then an external magnet is placed sequentially at four orthogonal directions to pull and push the magnetic particles in different directions, as shown in Figure 6a; four distinct colloidal structures are obtained by programming the magnetic field at the same electric field strength.

As observed above, when viewed from the top, wing structure formation in a droplet is accompanied by a transition between light shading and transparent states and this occurs in a reversible manner. Figure 6b presents the on and off cycle of a ≈350 µm droplet containing 8 wt% PS particles (*d* = 7 µm) driven at an AC field of *U* = 60 V_pp_ and *f* = 300 kHz. When the AC electric field is applied, the PS particles in the droplet quickly assemble thereby shifting from a light shading state to a transparent state after ≈0.5 s, while they retune to the light shading state within ≈3 s by actively pulling down the particles via a pDEP (Figure S10, Supporting Information). Without the pDEP force at the light shading state, the switching off states takes ≈40 s, being driven solely by gravity. Therefore, a bistable feature might also be possible by using density matching particles and a better optimized fluidic system. Moreover, the switching performance can be further accelerated by increasing the voltage, optimizing particle size (Figure S10d, Supporting Information), or decreasing the droplet size. The switching process is quite stable and is without obvious decay after undergoing more than 1000 cycles.

Figure 6c presents an integrated eMAP device designed with a SU‐8 grid layer upon the caterpillar electrodes. The electrode array is organized with a pitch of 365 µm and paired with a gap of 60 µm, matching the grid array, which has a pitch of 365 µm and side length of ≈350 µm. Droplets (*D* ≈ 350 µm) have been prepared using a capillary microfluidic device,^[^ ^38^ ^]^ which were then filled into the grid by the spreading effect of the oil phase^[^ ^39^ ^]^ and the DEP effect. When driven at *U* ≥ 60 V_pp_ and *f* = 300 kHz, the droplet array demonstrates synchronous switching performance, showing the colors of the substrate (white) and the particles (blue) at off and on states, respectively. Such a device can be programmed to selectively show different/contrast colors or grayscales as an optoelectronic display device. Furthermore, the switching time achieved in eMAP at this stage (≈0.5 s) is close to that of an electrophoretic display,^[^ ^19^ ^]^ and can theoretically be further reduced to 30 ms by scaling down the system (see the details in the Supporting Information). As the voltage is applied to the predesigned electrode pattern, a “heart” shape can be displayed by using a colored water phase (Figure 6d), demonstrating that the display color and the color contrast can be tuned via the applied electric field and the particle‐droplet materials.

Reliable technology for producing uniformly and stably distributed droplet arrays is required and is under development. The goal is to make a high‐throughput and large‐area optofluidic display device by integrating the eMAP array, which would enable not only the mass production of functional droplet/gel materials but also large‐area and high‐density optical switches/valves and reflective display devices as well.

## 3 Conclusion

We have proposed and validated an electro‐microfluidic assembly platform (eMAP) for programming particle assembly in water droplets that allows us to create and manipulate reconfigurable 3D colloidal assemblies in confined spaces. This eMAP seamlessly and sequentially combines dielectrophoretic droplet positioning and (di)electrowetting driven droplet deformation, promoting electric field penetration into water droplets, and thus facilitates dielectrophoretic induced colloid assembly in the water droplet. The deformed droplet confinement and the highly bent internal electric field lines enable the formation of colloidal assemblies which extend beyond the “near‐field” and “far‐field” cases. This offers the possibility to transport colloid particles to different spatial coordinates and form flexible colloidal structures. Overall, four main distinct 3D colloidal assemblies are observed, namely the bottom base (initial state), wing‐like, rising‐chain‐like, and top‐cap‐like structures. These structures can be flexibly tuned by the applied AC signals, as well as through variations to the properties of the particles and droplets. Programmable anisotropies with various patch compositions, shapes, sizes, and positions in a droplet can be produced by assembling pure or mixed particles/cells with different dielectric properties, sizes or densities. In addition, the integration of eMAP with colored water and particles has potential application in reflective color display devices. It is further conceived that these highly customizable colloidal building blocks can be expanded and mass‐produced by programming the predesigned electrode patterns and the microfluidic systems. Consistent and controllable assembly, except from colloidal particles, relies mainly on the droplet's uniformity and throughput which would be achievable by combining microfluidic and microelectronic techniques, being highly potential for advanced materials and electrical driven optofluidic devices.

## 4 Experimental Section

### eMAP

The design of the eMAP is shown in Figure 1a. Transparent indium‐tin oxide (ITO) coated glass slides were thoroughly cleaned by water bath ultrasound before use. The elliptical electrode array was fabricated using standard photolithography and wet‐etching technology in a Class 10 000 cleanroom. Caterpillar electrodes were designed with a pitch of 345 µm and a gap of 45 µm. A hydrophobic layer of Hyflon with a thickness of ≈900 nm was formed by spin‐coating a Hyflon solution (solid content of 2.5 wt%) onto the patterned ITO glass. The water droplets contained the colloidal dispersions. Silicone oil was selected as the outer phase to form water‐in‐oil emulsions. A 10 µL drop of the emulsion was pipetted directly onto the prepared solid surface. The water droplets in this work were mainly in the range of ≈90 to 690 µm, to satisfy the requirement that the droplets can contact at least two electrodes from neighboring rows. AC signals were applied to the ITO electrodes via a connected function generator (AFG 1062, Tektronix, Inc., USA) and an amplifier (ATA‐2042, Aigtek Co., Ltd, Xi'an, China), and monitored using an oscilloscope (TBS 1104, Tektronix, Inc., USA).

### Particles and Cells

Blue (*d*, 7 µm), yellow (*d*, 4 µm) and red colored (*d*, 7 µm) uniform PS microspheres were all purchased from Aladin (Shanghai, China). The PS particles (*d*, 10 and 15 µm) were purchased from Huge Biotechnology Co., Ltd. The PS particles (*d*, 20 µm) were purchased from DaE Science and Technology Co. Ltd. Silver‐coated hollow glass microspheres were bought from Cospheric LLC. Fe_3_O_4_@SiO_2_ particles (*d*, 2–3 µm) and Cu particles (*d*, ≈5 µm) were purchased from Aladin (China). SiO_2_ particles (diameters, 1 and 3 µm) were bought from NanJing Nanorainbow Biotechnology Co., Ltd. Yeast cells were purchased from Angel Yeast Co., Ltd (Yichang, China).

### Water‐in‐Oil Emulsion

DI water was prepared using an Ultrapure Water System (Water Purifier, Chengdu, Sichuan, China) with a resistivity of 18.25 MΩ cm. The silicone oil (50 cst) was purchased from Sigma‐Aldrich. After cleaning with DI water three times (centrifugation at 8000 rpm for 2 min), the colloid particles were dispersed in 50–200 µL DI water at the designated concentration. Then the water dispersion was sonicated for 30 min. To prepare the yeast cell dispersion, 4 g of dry yeast (Angel yeast Co., Ltd, China) was reactivated in 50 mL DI water at 35 °C for 1 h, and then washed with DI water three times at 2500 rpm for 1 min. Before each emulsification step, the colloid particles or yeast cells were resuspended by vortexing for 30 s to ensure a uniform concentration inside each droplet. 10 µL water dispersion was pipetted into 200 µL oil phase, and then emulsified using a Vortex Genie 2 (Scientific Industries, setting 4) for 10 s. A relatively low volume fraction of water of 5% was selected to obtain theexpected concentration and size of water droplets (surfactant‐free) after fusion. For the experiments conducted in the high throughput integrated eMAP, the surfactant KF‐6017 (0.5% v/v) was added in silicone oil to stabilize the formed water droplets.

### Numerical Simulations

COMSOL Multiphysics (Version 5.5) was employed to calculate the electric field distributions on the eMAP and within the water droplets. The electric potential satisfied Poisson's equation. The details of the simulation are shown in the Supporting Information.

### Data Acquisition and Analysis

The water droplets, colloidal structures, and their formation dynamics were directly observed and captured and recorded using a fluorescence inverted microscope (IX73, Olympus Co., Tokyo, Japan). The contact angle, side view of droplets and their inner structures were observed and analyzed with a contact angle measuring instrument. The aperture ratio data were obtained by analyzing the frames of the recorded videos of the colloidal structure formation dynamics using the Image J software.

## Conflict of Interest

The authors declare no conflict of interest.

## Author Contributions

S.S. and L.S. designed all experiments. S.S., X.Q., H.F., and S.X. performed the experiments. S.S., X.Q., M.J., and L.S. contributed to the data analysis as well as to the presentation of the results. S.S. and L.S. wrote and polished the initial manuscript, and Z.Y., M.J., G.Z., E.M.A., and P.M. contributed to the discussion and took part in writing towards the finalization of the manuscript.

## Supporting information

Supporting Information

Supplemental Movie 1

Supplemental Movie 2

Supplemental Movie 3

Supplemental Movie 4

## Data Availability

The data that support the findings of this study are available in the supplementary material of this article.
