# Supplementary material for: Electro‐Microfluidic Assembly Platform for Manipulating Colloidal Structures inside Water‐in‐Oil Emulsion Droplets
Source: Adv Sci (Weinh). 2022 Sep 28;9(32):2203341. doi: 10.1002/advs.202203341 (PMC9661862; doi:10.1002/advs.202203341)
Supplement: Supplementary file 1 — Supporting Information [file ADVS-9-2203341-s005.pdf]

ADVANCED SCIENCE
Open Access

Supporting Information

for *Adv. Sci.*, DOI 10.1002/advs.202203341

Electro-Microfluidic Assembly Platform for Manipulating Colloidal Structures inside Water-in-Oil Emulsion Droplets

*Shitao Shen, Xiaofeng Qin, Haoqiang Feng, Shuting Xie, Zichuan Yi, Mingliang Jin, Guofu Zhou, Eser Metin Akinoglu, Paul Mulvaney and Lingling Shui**

WILEY-VCH

ADVANCED SCIENCE
Open Access

Supplementary Information For:

Electro-microfluidic assembly platform for manipulating colloidal structures inside water-in-oil emulsion droplets

*Shitao Shen[1], Xiaofeng Qin[1], Haoqiang Feng[1], Shuting Xie[1], Zichuan Yi[1], Mingliang Jin[1], Guofu Zhou[1,3], Eser Metin Akinoglu[3,4], Paul Mulvaney[4], Lingling Shui[1, 2,] **

*Corresponding author. Email: shuill@m.scnu.edu.cn

The PDF file includes:

Figures S1-10

1. Optimization of electro-microfluidic assembly platform (eMAP).
2. Colloidal assemblies from the initial state of random distribution of colloids in water droplet.
3. Electrowetting performance of the water droplet on the substrate and the inner particle distribution in response to the frequency of the applied AC electric field.
4. Liquid environment in response to *U* and the top cap-like colloidal assembly behavior.
5. Demonstration of different assembly behaviors in droplets under various conditions.
6. Evolution of rising chain-like colloidal assembly
7. Proposed mechanism of the formation of colloidal assemblies.
8. Dielectrophoretic responses of particles (various sizes or dielectric properties) to frequency of the applied AC field.
9. Optical response corresponding to the colloidal assembly in droplets.

Supplementary Methods

Device fabrication and operation and **Simulation details**

Other Supplementary Material for this manuscript includes the following:

Movie S1 The dynamics and evolution of the spreading-wing-like structure in a ~210 μm water droplet containing 8 wt% PS particles (*d* = 7 μm) at *U* = 60 Vpp and *f* = 300 kHz.

Movie S2 The dynamics and evolution of the rising chain-like structure formation in a 476 μm water droplet containing 8 wt% PS particles (*d* = 7 μm) at *U* = 60 Vpp and *f* = 15 kHz.

Movie S3 The dynamics and evolution of the flytrap-like structure formation in a ~580 μm water droplet containing 8 wt% yeast cells (*d* = 5-7 μm) at *U* = 60 Vpp and *f* = 300 kHz.

Movie S4 The dynamics and evolution of a binary structure in a water droplet containing a spreading-wing-like structure of 6 wt% PS particles (*d* = 7 μm) and a tightly packed 6 wt% yeast cells (*d* = 5-9 μm) at *U* = 60 Vpp and *f* = 300 kHz.

1. Optimization of electro-microfluidic assembly platform (eMAP)

PS particles form colloidal chains along the electrical field lines, and the overlapping of chain clusters can be viewed from the top. The particle chains do not evolve smoothly on the conventional castle-shaped electrodes with either symmetric or offset alignments, as shown in **Figure S1a** and **b**. Therefore, to obtain ordered structures, a caterpillar electrode pattern comprising overlapped elliptical electrode arrays is used in this work.

The hydrophobic dielectric layer of Hyflon with thickness of ~900 nm is coated on the caterpillar electrodes to reduce the wettability of the electrode surface, allowing droplets to remain spherical shape. In addition, dielectric layer effectively prevents electrolysis and diminishes the electrohydrodynamic effect. The electric potential drops severely when passing across the dielectric layer and the surrounding oil. A small portion of the voltage drops near the bottom of the water droplet, as shown in **Figure S1c**. The water droplet confinement can distort its inner and outer local electric field distribution, meanwhile inducing highly curved electric field lines within it (**Figure S1d**). The quasi-circular electric field lines within the droplet are distinguished from the extensively investigated semicircular field[1].

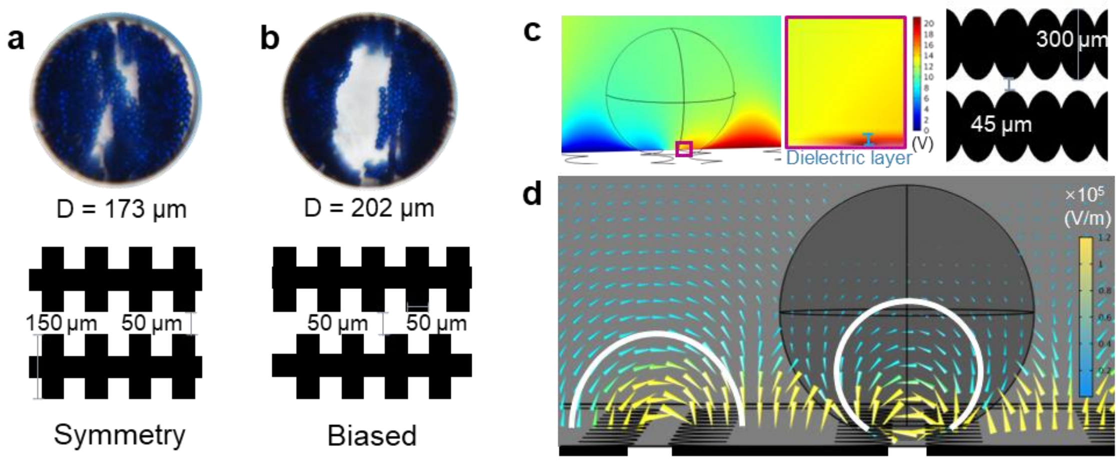

Figure S1. Colloidal assembly on castle-shaped electrode patterns with symmetric **a**) and offset **b**) alignments at 60 Vpp and 300 kHz. Simulated **c**) voltage distribution above elliptical electrode and **d**) highly curved electric field lines within the droplet. The simulations are all conducted at 60 Vpp and 300 kHz.

2. Colloidal assemblies from the initial state of random distribution of colloids in water droplet

When the W/O emulsion is prepared freshly, the inner colloid particles are dispersed randomly

within the water droplets. Starting from this state, the applied AC field can induce the assembly of particles into a mitotic spindle structure. When the electric field is turned off, the colloidal chains gradually disassemble and deposit (~70 s) at the bottom of the droplet.

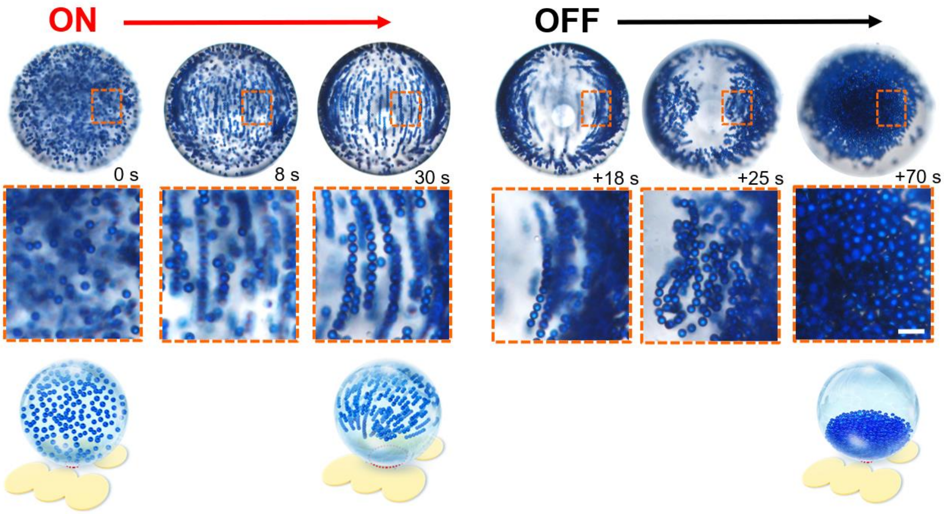

Figure S2. Assembling and disassembling 7 μm blue PS particles in a 396 μm water droplet as the AC electric field (U = 60 Vpp and f = 300 kHz) is switched ON and OFF. Scale bar, 20 μm. The solid content of the blue PS particles is 8 wt%. Schematics illustrate putative assembly positions.

3. Electrowetting performance of the water droplet on the substrate and the inner particle distribution in response to the frequency of the applied AC electric field

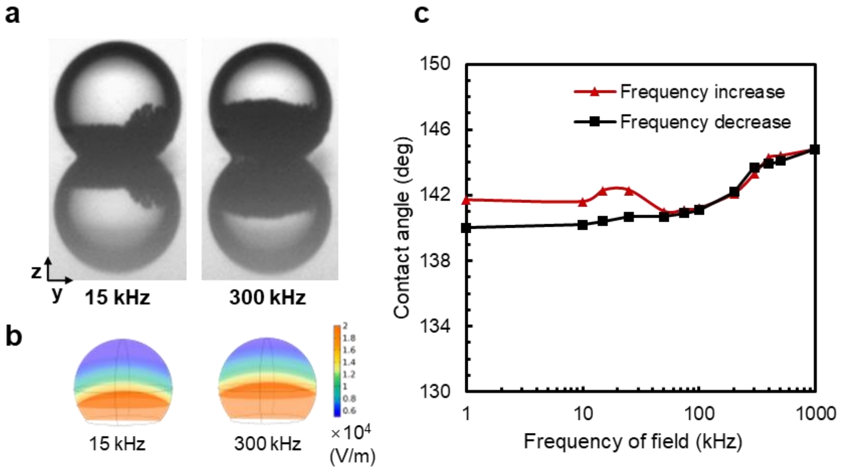

Figure S3. a) Side-view images of the inner particle assemblies and **b**) E-field distribution in a ~380 μm water droplet at f of 15 and 300 kHz, respectively, with U = 60 Vpp. **c**) Water droplet contact angle varying with f at U = 60 Vpp.

4. Liquid environment in response to *U* and the top cap-like colloidal assembly behavior

When *U* increases, in addition to the significant change of the assembly structure, the shape of the droplet and the flow environment inside the droplet will also vary. h_{particle} and *U* increase linearly, while the z-axis component of the electric field near the rising particles fluctuates slightly (**Figure S4a**). The droplets' shape also gradually changes slightly as *U* increases. As shown in **Figure S4b**, the deformation is smaller for the larger sized droplet.

As the particles elevate with increasing *U*, they eventually reach the ceiling of the droplet, forming a top-cap-like structure with particles closely packed in 2D hexagonal arrangement. The colloidal chains are clearly oriented along the y-axis just before merging into a 2D colloidal crystal near the ceiling of the droplet because of the electric field lines and the chain-chain interactions. Under strong nDEP conditions, the colloidal chains are squeezed, and eventually form 2D hexagonal close-packed crystalline structures within ~10 s (**Figure S4c**). We have counted the number of colloidal chains formed along the y-axis before they merge into 2D crystals, as shown in **Figure S4d** and **e**. Most particles (~40 %) prefer to form 49-56 μm long chains, meaning 7-8 particles per chain. In general, about 62% of particles can eventually form tightly packed crystalline structures. The normalized count shows the proportion of particles formed into chains with various chain lengths and the 2D crystals with various areas (the lengths and areas are both expressed in terms of the number of particles).

In our experiments, a flow field can be observed at high amplitude (>130 Vpp) and high frequency (>300 kHz) conditions due to the electrothermal effect (ACET). As *U* increases to 130 Vpp, *f* fixed at 300 kHz, the voltage amplitude at the bottom of droplet (U_{bottom}) is ~100 Vpp since 25% *U* drops at the dielectric layer. Therefore, the amplitude of *U* is 35 V_{RMS}. At this stage, droplet's shape can be seen as hemisphere under the dielectrowetting effect. Ignoring the effect of the surrounding oil phase and dielectric layer on droplet temperature transfer, the electrothermal effect which depends on the temperature gradient induced volume force, can be calculated from $\mathbf{F}_{\text{ACET}} = -M(\omega, T)\left(\frac{\varepsilon\sigma U_{\text{RMS}}^{4}}{2\text{k}\pi^{3}r^{3}T}\right)\left(1-\frac{2\beta}{\pi}\right)\widehat{\mathbf{n}_{\beta}}$, where *T* is the temperature, *r* is the distance between a certain point to the electrode center gap within droplet, and *β* is the angle between the line connecting this point and the center of the electrode gap and the horizontal direction, k = 0.6 J m^{-1} s^{-1} K^{-1}. A dimensionless factor that varies with *ω* and *T* is then written

as: $M(\omega, T) = (\frac{\frac{T\partial\sigma}{\sigma\partial T} - \frac{T\partial\varepsilon}{\varepsilon\partial T}}{1+(\omega\frac{\varepsilon}{\sigma})^2} + \frac{T}{2\varepsilon}\frac{\partial\varepsilon}{\partial T})$.

When the magnitude of the Coulomb force equals to the dielectric force, $M = 0$, the cross frequency can be calculated as: $\omega_c \sim \frac{\sigma}{\varepsilon}(2\frac{|\frac{\partial\sigma}{\sigma\partial T}|}{|\frac{\partial\varepsilon}{\varepsilon\partial T}|})^{0.5}$. For water, $\partial\sigma/\sigma\partial T$ = 2% per degree and $\partial\varepsilon/\varepsilon\partial T$ = -0.4%, so that the magnitude of $f_c = \omega_c (2\pi)^{-1}$ ~142 kHz in water droplet. The Coulomb force dominates at low frequencies ($f < f_c$) and the dielectric force dominates at high frequencies ($f > f_c$). Moreover, since the Coulomb force at low frequencies is ~10 times stronger than the dielectric force at high frequencies, the electrothermal effect is relatively weak at high frequency[1]. As f increases from 142 kHz (M = 0), electrothermal effect can gradually rise in our experiments. At f = 300 kHz, when U increases to 130 Vpp, particles experience different electrothermal effect at different positions. Assuming a droplet diameter of 350 μm, when particles reach the droplet's ceiling, they form a top cap-like structure at z of ~300 μm. The calculated particles velocity under DEP effect (v_{DEP}) is in the order of ~10 μm/s, and v_{ACET} is in the order of ~0.1-1 μm/s corresponding to the conductivity range of water. As a result, the weak electrothermal effect can only slightly change the orientation of the colloidal structure. When the particles are located at a lower position, the dielectrophoresis effect enhances more rapidly than the electrothermal effect, which can be described as: $\frac{v_{ACET}}{v_{DEP}} \propto \sigma U^2 \frac{r^2}{R^2}$. For instance, when a particle is near the floor of the droplet (z ~10 μm), v_{ACET} is in the range of about 10 to 100 μm/s, which is 10^3-10^4 orders less than v_{DEP} (~10^5 μm/s). Therefore, the electrothermal effect can be negligible when considering the assembly speed of the colloidal structures, while its effect on the assembly structure should be considered when the particle height $h_{particle}$ exceeds 300 μm at U > 130 Vpp and f > 300 kHz.

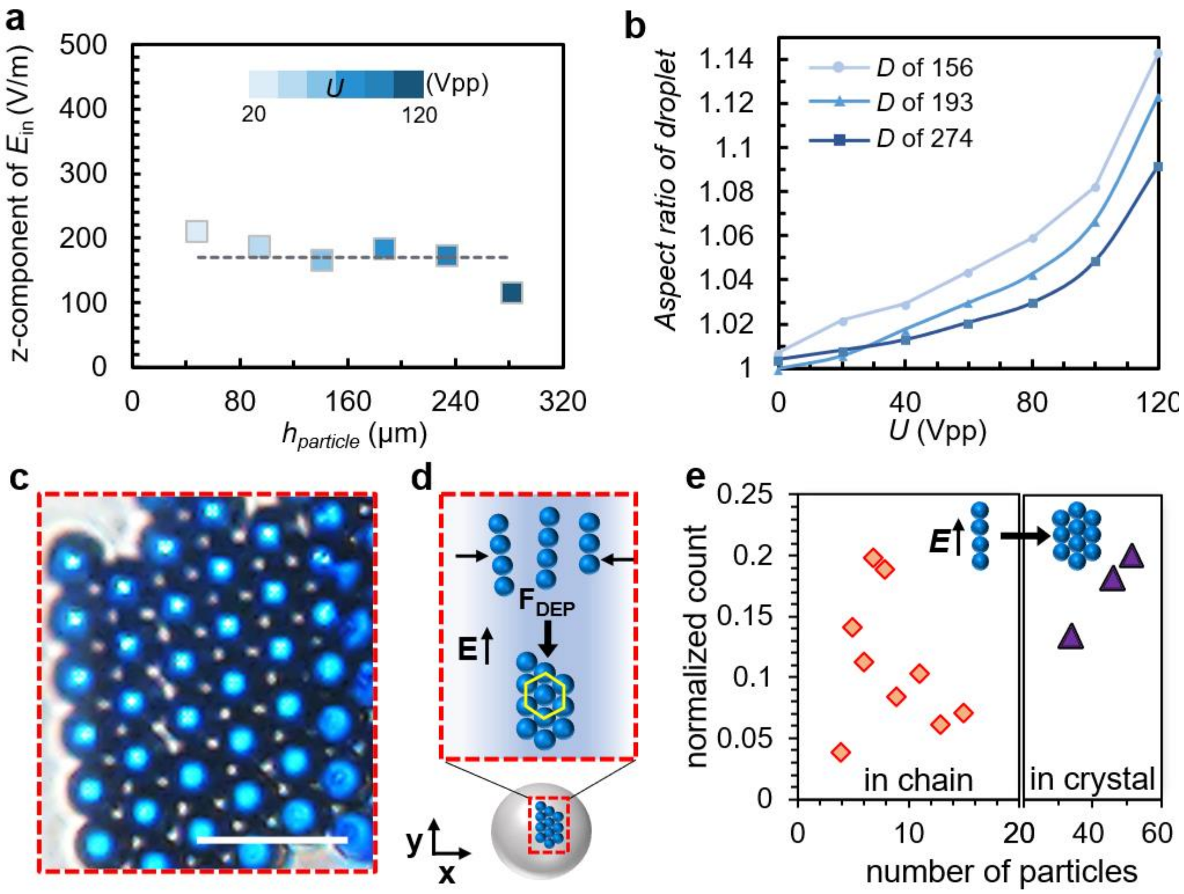

Figure S4. a) z-component of E_{in} distributed at wing structure positions varying with the simultaneous linear increase in U and $h_{particle}$. **b**) Length-to-width aspect ratio of droplets as a function of U for different sized droplets. The aspect ratio is quantified by the top-view images obtained in experiments. **c**) An optical image showing the detailed particle assemblies within the top cap-like structure in a 376 μm droplet at U = 130 Vpp and f = 300 kHz. Scale bar, 20 μm. **d**) Schematic illustration showing the colloidal chains assembling into crystalline structures. **e**) Normalized count of particles assembling into chains with various lengths or the 2D crystalline structure with different areas denoted by the number of particles in the same single layer. The droplet contains 1 wt% blue PS particles.

5. Demonstration of different assembly behaviors in droplets under various conditions

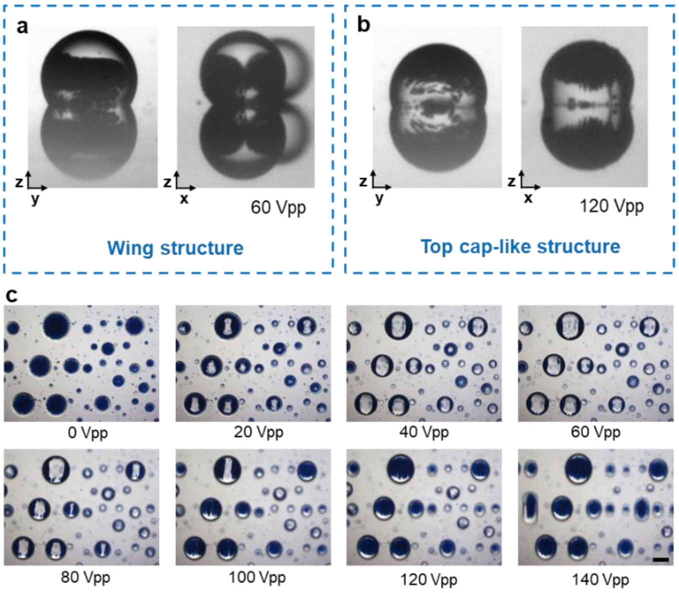

Figure S5. Orthogonal side views of the colloidal assembly structures formed in ~380 μm droplets at the applied electric fields of **a**) 60 Vpp and 300 kHz, and **b**) 120 Vpp and 300 kHz. **c**) Evolution of colloidal assembly structures versus *U* in various droplets at *f* = 300 kHz. Scale bar, 200 μm.

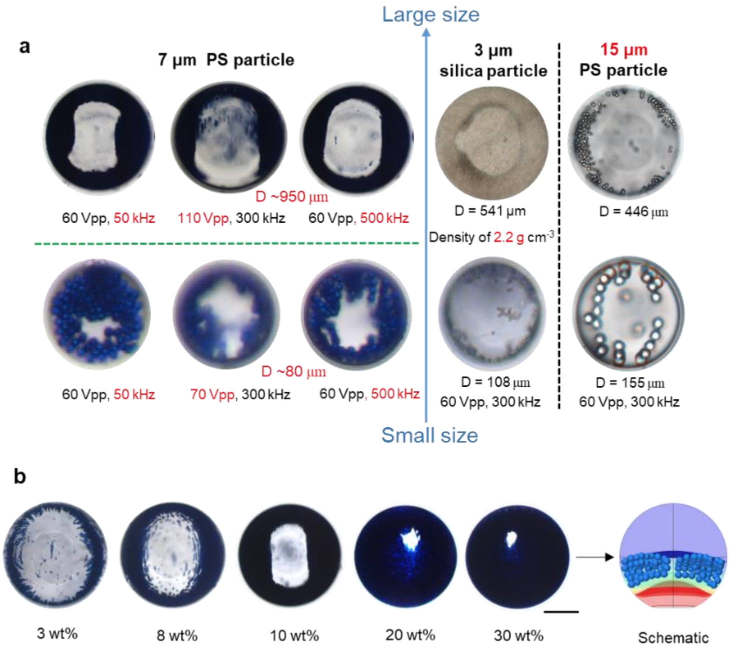

Figure S6. a) Various parameters range toward the assembly of wing-like structures. The solid content of all particles is 8 wt%. **b**) Colloidal structures forming at different solid content. Applied field at 300 kHz, 60 Vpp. Scale bar, 100 μm. Schematics illustrate putative assembly positions according to the electric field distribution.

6. Evolution of rising chain-like colloidal assembly

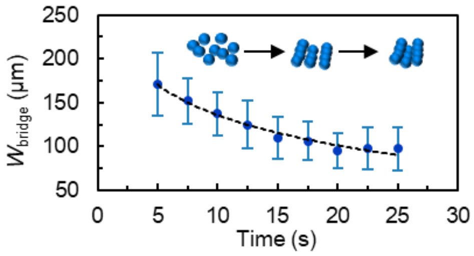

Figure S7. Width of the "bridging" chains varying with time at U = 60 Vpp and f = 15 kHz.

7. Proposed mechanism of the formation of colloidal assemblies

The majority of PS particles preferentially remain within the water droplets because of their wetting preference and image charge effects. The image charge effect is a strong, long-range (within 2 μm) force, possibly on top of a wetting effect[2]. In the case of a perfect spherical water droplet (ε_w) in an oil medium (ε_o), $\frac{\varepsilon_w}{\varepsilon_w+\varepsilon_o} \approx 1$, indicating that an image charge of the same sign appears at the mirror position. As a result, when approaching the droplet's interface, a charged particle may experience repulsive forces that increase with a decrease in the distance between the particle and the water-oil interface. Therefore, very few PS particles (less than 0.5 wt%) can be trapped at the interface. The particle trapping occurs due to the interfacial binding energy which can be calculated by $U_s = \pi R^2 \gamma_{ow} (1 - |\cos\theta'|)^2$, where R is the particle radius, $\gamma_{o\text{-}w}$ is the interfacial tension between water and silicone oil which is ~39 mN·m^{-1}, and θ' indicates the contact angle between the water-particle and the water-oil interfaces.

The potential energy of the particle dipole-dipole interaction can be given as: $U_{d\text{-}d} = \frac{1}{4\pi\varepsilon_0\varepsilon}(\frac{\vec{p}_1\cdot\vec{p}_2 - 3(\hat{n}\cdot\vec{p}_1)(\hat{n}\cdot\vec{p}_2)}{|\vec{r}_1 - \vec{r}_2|^3})$, where p is the electric dipole moment and $p_1 = p_2 = p = 4\pi\varepsilon_m f_{CM} R^3 E$ for the particles with the same properties, the length of vector $\vec{r}$ corresponds to the distance between the central points of two particles, $\hat{n}$ is the unit vector along the direction vector $\vec{r}$. θ is the angle between the vector $\hat{n}$ and the electric field direction. $U_{d\text{-}d} = \frac{p^2}{4\pi\varepsilon_0\varepsilon_m}\left(\frac{1-3\cos^2\theta}{r^3}\right)$, therefore $U_{d\text{-}d} \propto \frac{f_{CM}{}^2 R^6 E^2}{r^3}(1\text{-}3\cos^2\theta)$. The potential energy shows a positive correlation with the radius of particle and is proportional to E^2 and $f_{CM}{}^2$ which are frequency dependent. $U_{d\text{–}d}$ reaches zero when $\theta = 54.7^o$, indicating that $1 - 3\cos^2\theta = 0$. If the position of two particles is that at the angle $\theta > 54.7^o$, they will repel to each other; whereas when the angle $\theta < 54.7^o$, the attractive

interaction will occur. That is the reason why monodispersed particles inside droplets prefer to form single chain-like structures.

For the wing-like structures, the particles with induced dipole moments align parallel with electric field, therefore mostly forming colloidal chains along y-direction. The formed adjacent chains are aligned perpendicular at $\theta \sim 90^{o}$, as depicted in **Figure S8** (x-z plane), and some of them repel to each other, thus we can observe that the colloidal chains arrange themselves into feathery-like structures as shown in **Figure 3f**. Whereas, in certain cases, such as the top cap-like structure, the bridging chains and the rising chains can be squeezed into tightly packed structures subjected to the droplet interface confinement and the electric field direction effect. To evolve from wing-like structure to top cap-like structure, nDEP force renders particles to elevate along with the droplet interface, and finally squeezes the colloidal chains to form hexagonally close-packed crystalline structures at the ceiling of the water droplets.

The formation of rising chain-like structure can be divided into two quasi-orthogonal direction parts, the polarized particles "grow" vertically (with a slightly tilted angle) to the droplet interface above the electrode area and form "bridging" chains across the gap between the two electrodes. Particles grow vertically since the DEP force elevates a single particle and the particle-particle interactions cause colloidal chains to form along the electric field. From the top-view images shown in **Figure 4b**, we observe the unclose-packed colloidal chains array at the droplet interface above the electrode area. The length of the colloidal chain is observed to be in the range of 2*d* to 4*d* (*d*, particle diameter) and the chain-chain distance is observed to be *d* to 4*d*. This suggests a repulsive interaction between the vertically growing colloidal chains.

Within the same droplet, more interestingly, the "bridging" chains length is in the range of 5*d* to 12*d*, and tends to tighten up over time, suggesting the attractive chain-chain interaction. Chain-chain interactions can occur in the opposite direction due to the different colloidal chain lengths, which has been demonstrated in a simulation work[3]. The relative velocity of two adjacent chains can be attractive in the near field but repulsive in the far-field. At a critical distance, the transition from attractive to repulsive effect occurs as a function of the colloidal chain length. Thus, the longer chains are more likely to be subject to attractive interactions, which can explain the formation of the "bridging" chains.

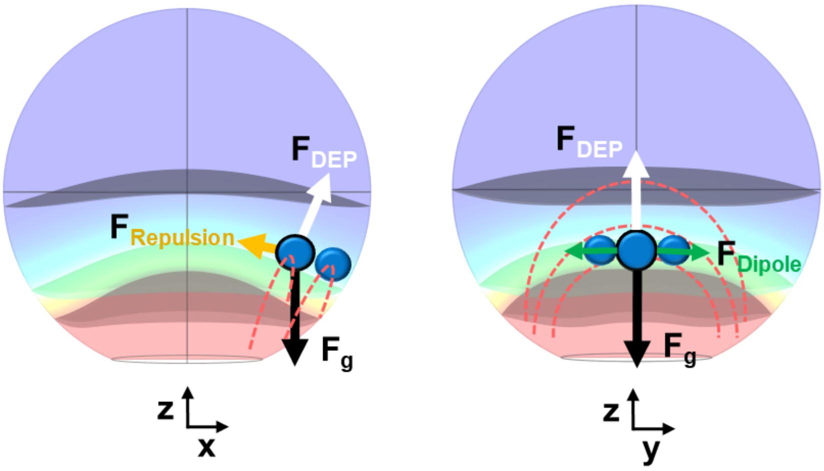

Figure S8. Two section views of the force analysis of the wing-like colloidal assembly structure at an AC electric field of U = 60 Vpp and f = 300 kHz.

8. Dielectrophoretic responses of particles (various sizes or dielectric properties) to frequency of the applied AC field.

The surface conductances of the PS and silica particles are 2.56 nS and 0.51 nS, respectively[4]. The relative permittivities of the PS and silica particles are set to 2.5 and 3.7, respectively. The Clausius-Mossotti factor of yeast cells is calculated using a double shell model[5]. The surrounding medium is deionized water, with permittivity of 80 and conductivity of 0.2 mS/m.

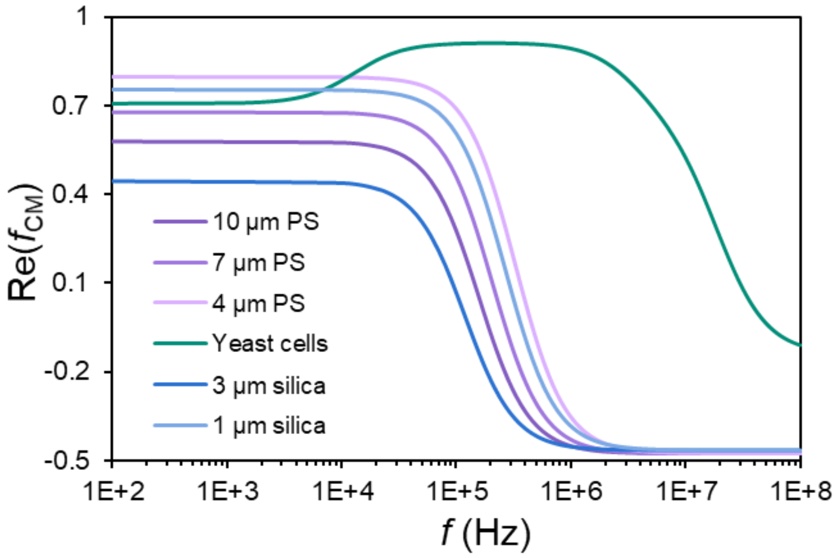

Figure S9. Calculated Re(f_{CM}) of PS particles (d of 4, 7, 10 µm), silica particles (d of 1, 3 µm) and yeast cells (d ~7µm) at the water conductivity of 0.2 mS/m.

9. Optical response corresponding to the colloidal assembly in droplets

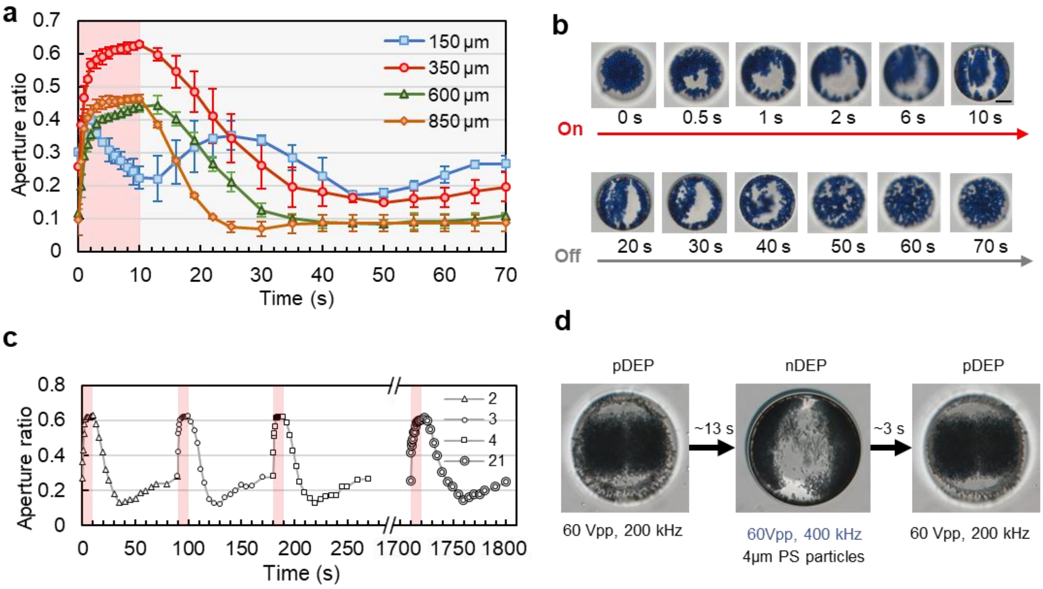

Figure S10. Optical response corresponding to the particle assembly in droplets. **a**) Top-view images of the open-close performance corresponding to the colloidal assemblies in the droplets with *D* of about 150, 350, 600 and 850 μm. The switching time is typically 10 s (On) and 60 s (Off), at *U* = 60 Vpp and *f* = 300 kHz. **b**) Top-view images showing the evolution of the particle assembles in a 150 μm droplet. The concentration of PS particles (*d* = 7 μm) is 8 wt%. Scale bar, 50 μm. **c**) Duration and reversibility of the particle assembly corresponding to **a**). **d**) pDEP and nDEP response of 8 wt% PS particles (*d* = 4 μm) in a water droplet achieved at different frequencies with *U* = 60 Vpp.

Programmable colloidal assembly in 3D droplet provides optical responsiveness at both micro- and macro-scales. As shown in **Figure S10a** and **Figure S10b**, a higher optical contrast is achieved in the 350 μm droplet compared to those with D of 150, 600 and 850 μm. The highest aperture ratio (calculated from the ratio of the opening area in the centre to the total area of the droplet viewed from the top) of 0.62 is achieved by applying an electric field of *U* = 60 Vpp and *f* = 300 kHz for 10 s. Moreover, to our surprise, we find that the larger droplets (brown and green curves) demonstrate a quicker "closing response" during the "folding wing-like" process compared to the smaller droplets. In addition, in small droplets, the particles reach the ceiling during the "On" stage and respond unexpectedly, for example in a 150 μm droplet as depicted in **Figure S10b**. The rapid "Off" stage can be achieved by tuning the field frequency. The repeatablility of assembly is evaluated as shown in **Figure 10c**. In addition, **Figure S10d**

demonstrates nDEP and pDEP reponses are achieved for the same 4 μm PS particles dispersed in water droplet at different frequencies.

In this work, the display performance is achieved according to the dielectrowetting facilitated dielectrophoresis, which can compensate to overcome some shortcomings of the conventional electrophoretic display (EPD) based on particle manipulation. Although the switching speed of 0.5s is close to the EPD device (mostly, being reported to be hundreds of milliseconds). However, in theory, the switching time could reach ~30 ms by minimizing the electrode gap (e.g. 25 μm) and reducing the droplet's size to ~50 μm. Moreover, the achievable modes are variable, enabling an additional light-transmitting state due to the "wing structure", thus can reduce the applied particle types within a droplet/encapsule and simplify the driving system. In addition, based on the working principle and achieved results, the eMAP is adaptive to various types and sizes of colloidal particles, and thus highly simplify the preparation of colored particles for display application.

Supplementary Methods

Device Fabrication and Operation

High throughput integrated eMAP consists of Hyflon coated ITO electrode and a photoresist (Microchem, SU-8 2075) grid layer on it. The grid layer was designed with a pitch of 365 μm and side length of ~350 μm. To match with the grid layer, electrode pattern was also organized with a pitch of 365 μm and paired with a gap of 60 μm. Firstly, the ITO-glass was cleaned and patterned to the designed structures using a photolithography technology. A layer of Hyflon was then coated on to the onto the patterned electrode surface to form a hydrophobic insulating layer. Afterwards, the Hyflon was plasmonically treated to be hydrophilic before spin-coating a SU-8 (photoresist) layer (900 rpm, 65 s). A second photolithography process was applied to form the grid on the Hyflon coating. The obtained substrate was hard baked at 120°C for 5min to obtain the high throughput eMAP device.

For the experiments conducted in the high throughput integrated eMAP, the surfactant KF-6017 (0.5 % v/v) was added in silicone oil to stabilize the formed water droplets. The monodisperse droplets were generated by using a capillary microfluidic device, which were then filled to the grid cell by the spreading effect of the oil phase and the DEP effect. The electric field was applied via the standard electric interconnectors and voltage supplier. And all the rest procedures were the same as those for manipulating a single eMAP.

Simulation details

A finite element simulation using electric current model was performed to investigate the electric field distribution using COMSOL Multiphysics 5.5. The electric potential satisfies Gauss's law. By using the frequency domain capability within the software, the electric field calculation combines the time-harmonic equation and Gauss's law:

$$\nabla \cdot J = \nabla \cdot (\sigma \mathbf{E} + J_e) = -j\omega\rho$$

$$\nabla \cdot \varepsilon E = \rho$$

$$\mathbf{E} = -\nabla U$$

$$U = \text{Amplitude} \times \cos(\omega t)$$

where J and J_e are the current density and externally generated current density, respectively, ρ is the space charge density, U is the electric potential, and U_{RMS} is the mean square root (RMS) of the electric potential. **E** is the electric field, ω is the angular frequency, t is the time, and n denotes the unit normal vector pointing into electrolyte, while σ and ε are the conductivity and permittivity, respectively.

The geometry of the 3D model was built based on the proposed eMAP. A box of $1.6 \times 1.6 \times 1.0$ mm^3 is set as the oil phase with a relative permittivity of 2.7 and a conductivity of 1×10^{-7} mS m^{-1}.The box contains quasi-spherical droplets with diameters, D, of 360 and 460 µm, corresponding to the results shown in **Figures. 2** and **4**. The conductivity and relative permittivity of the aqueous droplet is set at 0.2 mS m^{-1} and 80, respectively. The thickness of the hydrophobic dielectric layer is set at 900 nm with a relative permittivity of 2.0 and a conductivity of 1×10^{-7} mS m^{-1}. The mesh of the model consists of triangles (surface) and tetrahedra (volume). The boundary conditions for the electric field are:

In the bulk: $\nabla^2 U = 0$, $\mathbf{E} = -\nabla U$.

At the AC signal applied electrode: $U = 30 \times \cos(\omega t)$, $U_{RMS} = 21.2$ V.

At the grounded electrodes: $U = 0$ V.

At the wall: $\mathbf{n} \cdot \nabla U = 0$.

References

[1] A. Ramos, H. Morgan, N. G. Green, A. Castellanos, *Journal of Physics D: Applied Physics*

1998, 31, 2338.
[2] a)M. E. Leunissen, A. Van Blaaderen, A. D. Hollingsworth, M. T. Sullivan, P. M. Chaikin, *Proceedings of the National Academy of Sciences* **2007**, 104, 2585; b)H. von Grünberg, E. Mbamala, *Journal of Physics: Condensed Matter* **2001**, 13, 4801.
[3] J. S. Park, D. Saintillan, *Physical Review E* **2011**, 83, 041409.
[4] C.-K. Yeh, J.-Y. Juang, *AIP Advances* **2017**, 7, 065304.
[5] C. Lyu, J. Wang, M. Powell-Palm, B. Rubinsky, *Scientific reports* **2018**, 8, 1.
